# Cyclovirobuxine D Inhibits Cell Proliferation and Induces Mitochondria-Mediated Apoptosis in Human Gastric Cancer Cells

**DOI:** 10.3390/molecules201119729

**Published:** 2015-11-19

**Authors:** Jie Wu, Zhujun Tan, Jian Chen, Cheng Dong

**Affiliations:** 1 School of Naval Architecture, Ocean and Civil Engineering, Shanghai Jiao Tong University, No. 800 Dongchuan Road, Shanghai 200240, China; cxdbio@engr.psu.edu; 2 Ministry of Education of China (MOE) Key Laboratory of Hydrodynamics, Shanghai Jiao Tong University, No. 800 Dongchuan Road, Shanghai 200240, China; 3 Department of General Surgery, School of Medicine, Xinhua Hospital, Affiliated to Shanghai Jiao Tong University, Shanghai 200092, China; tanzj1981@163.com; 4 School of Pharmacy, Shanghai Jiao Tong University, No. 800 Dongchuan Road, Shanghai 200240, China; chenjian@sjtu.edu.cn; 5 Department of Biomedical Engineering, The Pennsylvania State University, University Park, PA 16802, USA

**Keywords:** cyclovirobuxine D, cell cycle, gastric cancer, apoptosis, mitochondria

## Abstract

Gastric cancer is one of the most common malignant cancers, with high death rates, poor prognosis and limited treatment methods. Cyclovirobuxine D (CVB-D) is the main active component of the traditional Chinese medicine *Buxus microphylla.* In the present study, we test the effects of CVB-D on gastric cancer cells and the underlying mechanisms of action. CVB-D reduced cell viability and colony formation ability of MGC-803 and MKN28 cells in a time- and concentration-dependent manner. Flow cytometry showed that cell cycle of CVB-D treated cells was arrested at the S-phase. CVB-D also induced apoptosis in MGC-803 and MKN28 cells, especially early stage apoptosis. Furthermore, mitochondria membrane potential (Δψm) was reduced and apoptosis-related proteins, cleaved Caspase-3 and Bax/Bcl-2, were up-regulated in CVB-D-treated MGC-803 and MKN28 cells. Taken together, our studies found that CVB-D plays important roles in inhibition of gastric tumorigenesis via arresting cell cycle and inducing mitochondria-mediated apoptosis, suggesting the potential application of CVB-D in gastric cancer therapy.

## 1. Introduction

Gastric cancer is one of the most widespread cancers and the second leading cause of cancer-related death in the world [1,2]. It is diagnosed more frequently in 55-year or older males than other populations. The prognosis of gastric cancer is still poor and its cause remains unknown. So far, surgery is effective to cure most of patients with early stage gastric cancer. However, more than half of the patients with advanced stage of gastric cancer die of locoregional relapse and distant metastases after surgery [2,3]. Thus, there is an urgent need to develop novel active drugs to prevent and treat gastric cancer.

Cyclovirobuxine D (CVB-D, 3β,5α,16α,20*S*)-4,4,14-trimethyl-3,20-bis(methylamino)-9,19-cyclo-pregnan-16-ol, C_26_H_46_N_2_O, molecular weight 402.662), is an alkaloid component extracted from the root of *Buxus microphylla,* a traditional Chinese medicine. For hundreds of years, people in China have been using *Buxus microphylla* to treat/prevent various cardiovascular diseases [4,5]. CVB-D, as the main active component of *Buxus microphylla*, has been proved in both laboratory and clinical studies to have beneficial effects on heart failure, arrhythmias, myocardial ischemia and other cardiovascular diseases of humans and other animals [4,5,6,7,8,9,10,11]. Recent studies have also found that CVB-D exerts broader beneficial effects on human diseases other than cardiovascular dysfunction, such as cancer. Lu *et al.* showed that CVB-D could induce autophagy-associated cell death via the Akt/mTOR pathway in human breast cancer cells [12]. However, whether and how CVB-D affects other cellular processes and the tumorigenesis pathway of cancer cells is still largely unknown. In the present study, we investigated the effects of CVB-D on human gastric cancer cells, particularly its roles in inducing apoptosis. Our studies are expected to shed light on the biological activities of CVB-D in cancer.

## 2. Results

### 2.1. CVB-D Reduces Cell Viability and Colony Formation Ability of Gastric Cancer Cells

**Figure 1:**
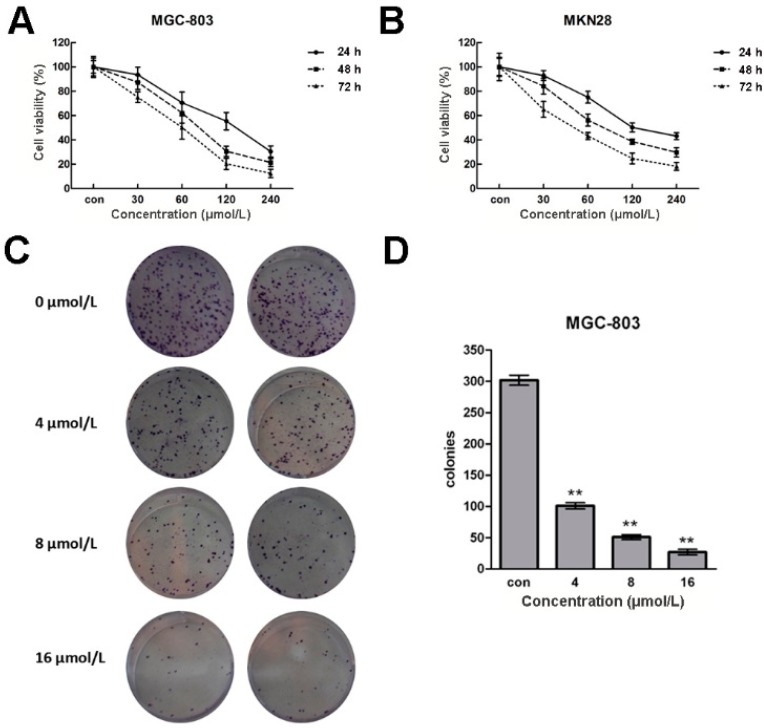

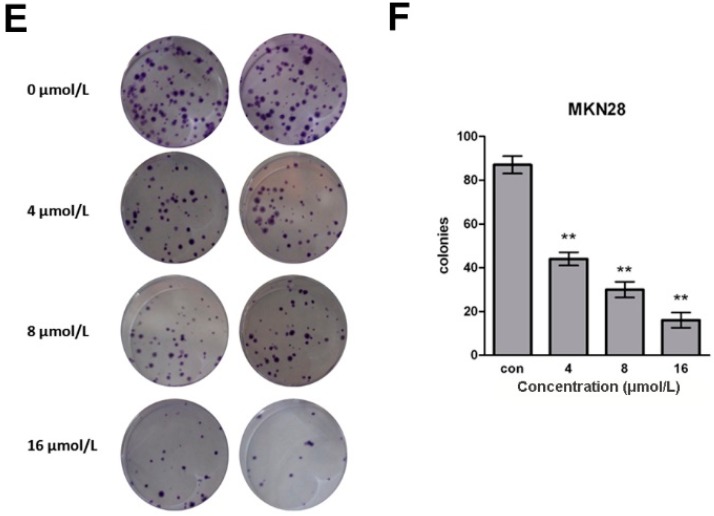
CVB-D induces cell viability of MGC-803 and MKN28 cells. (**A**,**B**) MTT assays of cell viability of MGC-803 (**A**); and MKN28 cells (**B**) at 24, 48 and 72 h after treatment with CVB-D (0, 30, 60, 120 and 240 µmol/L). Each experiment involved at least three replicates; (**C**,**E**) Representative images of crystals violet staining assays of CVB-D (0, 4, 8 and 16 µmol/L) treated MGC-803 (**C**); and MKN28 cells (**E**); (**D**,**F**) Colony numbers of CVB-D treated MGC-803 (**D**); and MKN28 cells (**F**). ** *p* < 0.01. Each experiment involved at least three replicates.

To study the potential role(s) of CVB-D in gastric cancer cells, we firstly tested the cell viability of MGC-803 and MKN28 cells after CVB-D treatment. After incubation with 0, 30, 60, 120 and 240 µmol/L CVB-D for 24, 48 and 72 h, the viabilities of MGC-803 and MKN28 cells were measured using an MTT assay. As shown in Figure 1A,B, both cell lines showed a concentration- and time-dependent reduced cell viability after CVB-D treatment. Only ~10% MGC-803 cells and 20% MKN28 cells were alive at 72 h after treatment with 240 µmol/L CVB-D, compared with untreated cells.

Next we analyzed the colony formation ability of MGC-803 and MKN28 cells after CVB-D (0, 4, 8 and 16 µmol/L) treatment. As shown in Figure 1C–F, crystal violet staining indicated that the colony numbers of CVB-D-treated MGC-803 and MKN28 cells were decreased dramatically compared with untreated cells. There were only 1/10 colonies detected in 16 µmol/L CVB-D-treated MGC-803 cells. The above results suggest that both gastric cancer cell viability and colony formation ability are reduced in response to increased concentrations of CVB-D.

### 2.2. CVB-D Arrests Cell Cycle Progression of Gastric Cancer Cells

**Figure 2:**
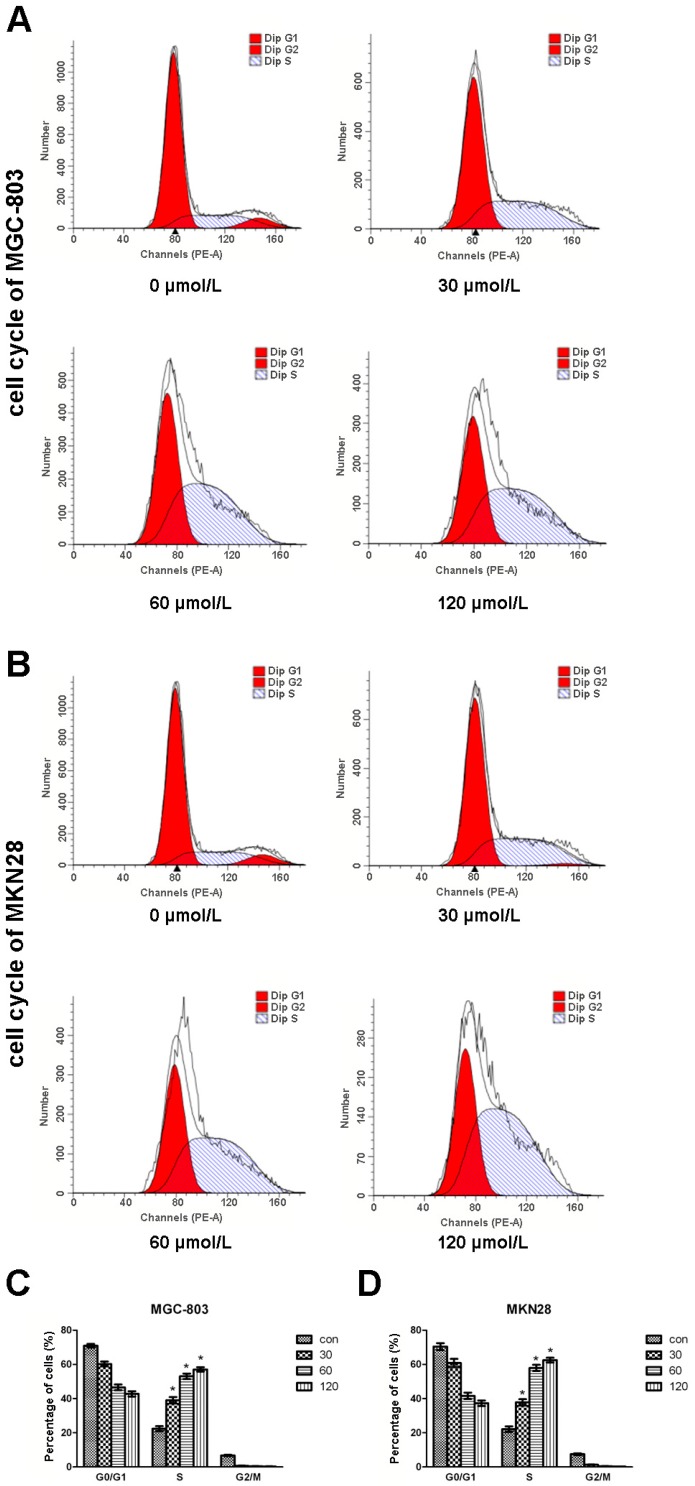
CVB-D arrests cell cycle progressions of MGC-803 and MKN28 cells. (**A**,**B**) Representative graphs of flow cytometry analysis of cell cycle stages of CVB-D (0, 30, 60 and 120 µmol/L) treated MGC-803 (**A**); and MKN28 cells (**B**); (**C**,**D**) Statistic analysis of cells numbers at G_0_/G_1_, S and G_2_/M stages of CVB-D treated MGC-803 (**C**); and MKN28 cells (**D**). * *p* < 0.05. Each experiment included at least three replicates.

The cell cycle plays key roles in cancer cell proliferation. We therefore analyzed the cell cycle of CVB-D-treated MGC-803 and MKN28 cells using flow cytometry. As shown in Figure 2, more cells were arrested at S phase compared with untreated cells, while cell numbers at the other two populations were both decreased. This effect of CVB-D on cell cycle was concentration-dependent. The percentages of cells at S phase of 120 µmol/L CVB-D-treated MGC-803 and MKN28 cells were ~3-fold that of untreated cells. These results indicated that CVB-D could arrest the cell cycle of gastric cancer cells at S phase in a concentration-dependent manner, which might contribute to reduced cell growth and colony formation.

### 2.3. CVB-D Leads to Apoptosis of Gastric Cancer Cells

**Figure 3:**
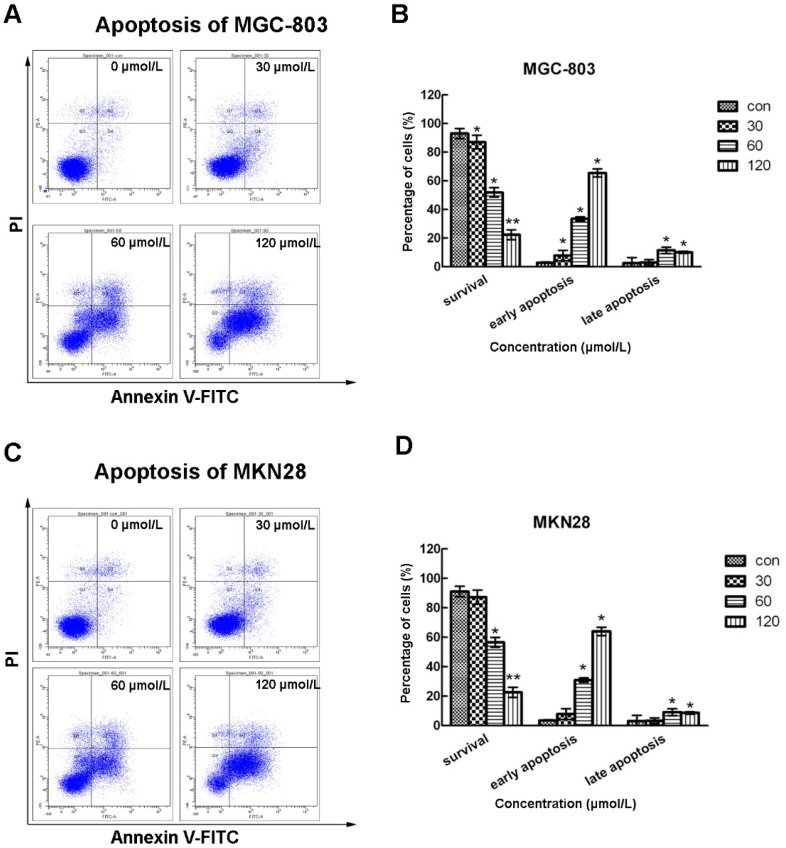
CVB-D induces apoptosis of MGC-803 and MKN28 cells. (**A**,**C**) Representative flow cytometry graphs indicating apoptosis patterns of CVB-D (0, 30, 60 and 120 µmol/L) treated MGC-803 (**A**); and MKN28 cells (**C**); (**B**,**D**) Statistic analysis of cells numbers at different populations of CVB-D treated MGC-803 (**B**); and MKN28 cells (**D**). PI^−^/Annexin V^−^, survival cells; PI^−^/Annexin V^+^, early apoptotic cells; PI^+^/Annexin V^+^, late apoptotic cells. * *p* < 0.05, ** *p* < 0.01. Each experiment involved at least three replicates.

Apoptosis might be another reason which causes inhibited gastric cell growth and colony formation. To test this prediction, we stained CVB-D treated MGC-803 and MKN28 cells using PI and Annexin V dyes and analyzed them by flow cytometry. As shown in Figure 3, surviving cells were PI^−^/Annexin V^−^, early apoptotic cells were PI^−^/Annexin V^+^ and late apoptotic cells were PI^+^/Annexin V^+^. Most of the untreated MGC-803 and MKN28 cells (>90%) were surviving cells. However, with increased concentrations of CVB-D, the numbers of surviving cells were decreased remarkably, while many more apoptotic cells were detected, especially early apoptotic cells. Only ~20% MGC-803 and MKN28 cells treated with 120 µmol/L CVB-D were still alive and more than 60% MGC-803 and MKN28 cells were at the early apoptotic stage. Thus, CVB-D leads to apoptosis in gastric cancer cells in a concentration-dependent manner, which might result in reduced cell growth and colony formation.

### 2.4. CVB-D Results in Loss of ΔΨm in Gastric Cancer Cells

**Figure 4:**
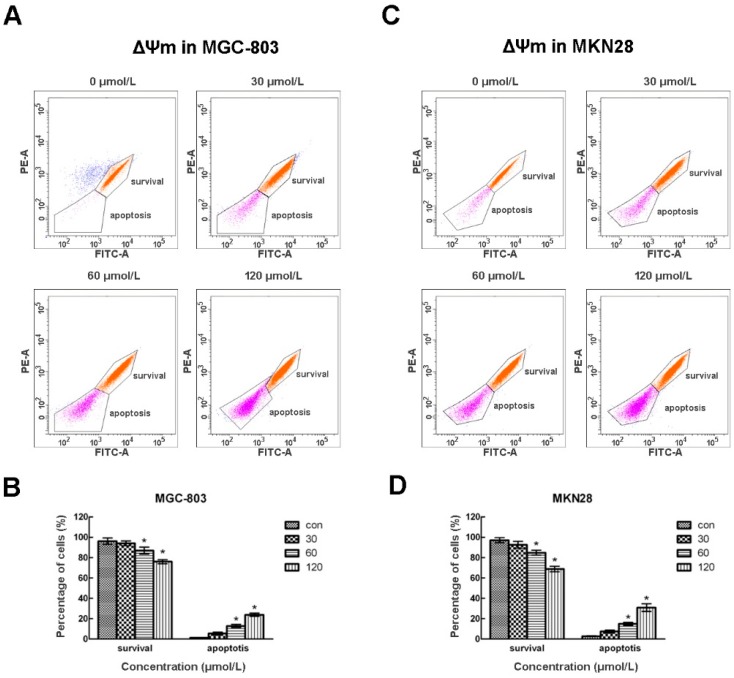
CVB-D results in loss of ΔΨm in MGC-803 and MKN28 cells. (**A**,**C**) Representative flow cytometry graphs of ΔΨm of CVB-D (0, 30, 60 and 120 µmol/L) treated MGC-803 (**A**); and MKN28 cells (**C**); (**B**,**D**) Statistic analysis of cells numbers at different populations of CVB-D treated MGC-803 (**B**); and MKN28 cells (**D**). Rh123^+^, Survival cells; Rh123^−^, apoptotic cells. * *p* < 0.05. Each experiment involved at least three replicates.

Mitochondrial dysfunction has been shown to participate in the induction of apoptosis, so we stained the CVB-D treated cells using the fluorescent dye Rh123 which could stain intact mitochondria, and performed flow cytometry to determine its effects on mitochondrial membrane integrity. As shown in Figure 4, Rh123 staining divided the cells into two populations. Cells with high Rh123 signals were surviving cells and cells with low Rh123 signals were apoptotic cells. Most untreated MGC-803 and MKN28 cells showed high Rh123 signals, while there was a concentration-dependent increase of apoptotic cells with low Rh123 signals. These results suggest that CVB-D treatment caused mitochondria membrane leakage and loss of ΔΨm in gastric cancer cells, thus inducing a mitochondria-dependent pathway of apoptosis in gastric cancer cells.

### 2.5. CVB-D Induces Expression of Apoptosis-Related Proteins in Gastric Cancer Cells

**Figure 5:**
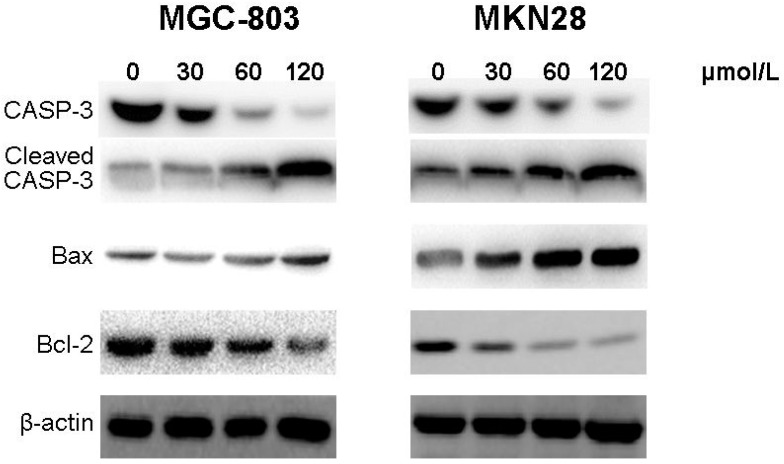
CVB-D induces expression of apoptosis-related proteins in MGC-803 and MKN28 cells. The protein bands of cleaved Caspase-3, Bax and Bcl-2 were detected using western blotting in CVB-D (0, 30, 60 and 120 µmol/L) treated MGC-803 (**left**); and MKN28 cells (**right**). β-actin was used as the control protein.

We then analyzed the expression of apoptosis-related proteins by western blotting. As shown in Figure 5, cleaved Caspase-3 and Bax were both up-regulated while the expression of Bcl-2 was decreased in CVB-D-treated MGC-803 and MKN28 cells in a concentration-dependent manner. 

These results indicated that CVB-D might cause apoptosis via up-regulation of the apoptosis- related proteins, cleaved Caspase-3 and ratio of Bax/Bcl-2, in gastric cancer cells.

## 3. Discussion

Gastric cancer is one of the most lethal malignancies and the second leading cause of cancer-related deaths in the world. Although surgery is effective to cure most of patients with early stage gastric cancer, more than half of the patients with advanced stage of gastric cancer still die of relapse and distant metastases after surgery. Thus, it is important to develop novel active drugs to prevent and treat gastric cancer.

CVB-D is well known as an active alkaloid of a traditional Chinese medicine used to prevent and/or treat many cardiovascular diseases [10]. It has been reported that CVB-D is also frequently used to treat a variety of human hematological and solid tumors, such as leukemia and breast cancer. However, it is still unknown whether CVB-D could be applied on treatment of gastric cancer and how CVB-D affects the tumorigenesis pathway of gastric cancer cells. In the current study, we found CVB-D could inhibit human gastric carcinoma growth and induce mitochondria-mediated apoptosis of gastric carcinoma cells.

The MTT assay showed that CVB-D decreased both MGC-803 and MKN28 cell viability in a concentration- and time-dependent manner, which was accompanied with impaired colony formation. Lu *et al.* found the similar effects of CVB-D on MCF-7 human breast cancer cells. However, in human breast cells autophagy associated cell death caused by CVB-D led to cell growth inhibition [12], while the main reasons for reduced cell viability in CVB-D gastric cancer cells were arrested cell cycle and induced mitochondria-mediated apoptosis. Whether autophagy associated cell death is also involved in the CVB-D induced gastric carcinoma cell growth inhibition need further investigation.

A previous study found CVB-D could block the JAK-STAT signaling pathway [13], which transmits information from chemical signals outside the cell to gene promoters on the DNA in the nucleus and regulates gene transcription. The JAK-STAT signaling pathway has been shown to be involved in oncogenesis through modulation of gene expression [14,15]. Evidence shows that some genes regulated by the JAK-STAT signaling pathway are related with various cellular processes, such as cell proliferation and apoptosis [15,16,17,18,19,20]. Cell apoptosis is an autonomous cell death process, which can be induced by a variety of drugs and physical and chemical factors. The key players in this cellular process is the family of cysteine-containing aspartate-specific proteases (caspase) which contains many members. Caspase-3, which is one of the most important members of this protein family has been reported to be the key executor of cell apoptosis. When activated by the external apoptosis signals, caspase-3 can be activated and induce the apoptosis signaling pathway of the cells by the interaction with many other proteases. In our research, we demonstrated that CVB-D might cause apoptosis via up-regulation of the apoptosis related proteins, such as cleaved Caspase-3 and ratio of Bax/Bcl-2, in gastric cancer cells. It would be interesting to investigate in future studies whether CVB-D-induced apoptosis of gastric cancer cell occurs through modulation of the gene expression profile of carcinoma cells.

In myocytes, CVB-D has been found to be involved in the regulation of the expression of several calcium cycling proteins thus affecting calcium levels inside cells [21]. Calcium mobilization plays key roles in regulating several signaling networks controlling tumor cell growth, differentiation, or apoptosis [22,23,24], so CVB-D might inhibit gastric cancer cell death and induce apoptosis via calcium regulation. Testing this hypothesis would help us learn in more detail about the mechanism of action of CVB-D in the inhibition of tumorigenesis.

## 4. Experimental Section

### 4.1. Cell lines and Culture Conditions

Gastric cancer cell lines MGC-803 and MKN28 were acquired from Cell Bank of Chinese Academy of Sciences (Shanghai, China). These cells were cultured in Dulbecco’s RPMI1640 (Gibco, Grand Island, NY, USA) supplemented with 10% fetal bovine serum (FBS; Gibco, Grand Island, NY, USA) and penicillin-streptomycin (Hyclone, Logan, UT, USA) at 37 °C in a humidified atmosphere of 5% CO_2_.

### 4.2. MTT Assay

Cell proliferation of MGC-803 and MKN28 cells was monitored by a 3-(4,5-dimethylthiazol-2-yl)-2,5-diphenyltetrazolium bromide (MTT). In brief, the cells were treated with 0, 30, 60, 120 and 240 µmol/L CVB-D for 24, 48 and 72 h, respectively. Then we added MTT solution into cells and continued the incubation at 37 °C for additional 3 h. After adding acidic isopropanol, the optical density (OD) of each well was measured at a wavelength of 590 nm.

### 4.3. Crystal Violet Staining

MGC-803 and MKN28 cells were cultured in complete medium supplemented with agarose at a density of 400 cells/well in 6 well plates. After incubation for 48 h, the cells were then cultured in media with 0, 4, 8 and 16 µmol/L CVB-D at 37 °C with 5% CO_2_. The cells were then fixed with 4% PFA and stained with 0.1% crystal violet and analyzing using a microscope.

### 4.4. Flow Cytometry

MGC-803 and MKN28 cells were seeded in 6-well plate and treated with 0, 30, 60 and 120 µmol/L CVB-D for further analysis. For cell cycle analysis, the gastric cells were treated with different concentrations of CVB-D for 48 h and then washed in cold PBS and fixed with pre-cold 75% ethanol. Then the cells were digested using RNase (10 mg/mL) and stained with propidium iodide (PI, 1 mg/mL). To analyze apoptosis, the cells were incubated with different concentrations of CVB-D for 48 h and then washed in cold PBS and stained with Annexin V-FITC (100 μg/mL) for 15 min in the dark. After centrifuge at 3000 rpm for 3 min, the cells were resuspended in PBS. To measure mitochondrial membrane potential (∆Ψm), cells were incubated with different concentrations of CVB-D for 48 h and then washed twice with PBS and stained with Rh123 (10 μg/mL). After incubation at 37 °C for 30 min in the dark, the cells were collected and resuspended in PBS. All the stained cells were analyzed immediately by flow cytometry.

### 4.5. Western Blotting

MGC-803 and MKN28 cells were subcultured in a 6-well plate. After incubation for 24 h, the cells were treated with 0, 30, 60 and 120 µmol/L CVB-D for 48 h. Cells were then washed with ice-cold PBS and lysed in lysis buffer (Beyotime, Shanghai, China). 40 mg proteins were applied to 10% SDS-polyacrylamide gel. After electrophoresis, the proteins were transferred to PVDF membranes, which were then blocked in 1% bovine serum albumin (BSA) in TBST for 1 h. The membranes were probed with primary antibody for cleaved caspase 3 (1:1000; Cell Signaling Technology, Beverly, MA, USA), Bax (1:1000; Cell Signaling Technology), Bcl-2 (1:1000; Cell Signaling Technology) and β-actin (1:1000; Cell Signaling Technology) at 4 °C overnight. Then the membranes were washed and incubated with horseradish peroxidase conjugated goat anti-mouse (1:5000; Abcam, Cambridge, UK) and goat anti-rabbit (1:5000; Abcam) secondary antibodies for 2 h. The membrane was washed and visualized using Chemiluminescent ECL reagent (Pierce, Rockford, IL, USA).

### 4.6. Statistical Analysis

Statistical differences between two groups were determined using Student’s *t* test. The differences were considered statistically significant at *p* < 0.05. Data are presented as mean ± standard deviation (SD).

## 5. Conclusions

To the best of our knowledge, this is the first study to reveal the effects of cyclovirobuxine D (CVB-D), an active alkaloid of a traditional Chinese medicine, to induce apoptosis in gastric cancer. To summarize, our present studies have proved that CVB-D inhibits cell proliferation and colony formation of gastric cancer cells through suppression of cell cycle progression and inducement of mitochondria-mediated apoptosis. The findings of our present work provide good evidence for the anti-cancer effect of (CVB-D) and suggest that CVB-D is a good potential compound for gastric cancer treatment.
